# Targeting BMP and TAZ/TEAD mechanotransduction pathways impairs acute myeloid leukemia chemoresistance

**DOI:** 10.1038/s41375-026-02904-7

**Published:** 2026-03-18

**Authors:** Léa Barral, Nicolas Lespinasse, Camila Martin Cardozo, Sandrine Jeanpierre, Anna Bourgeois, Katharina Rösel, Emmanuel Beillard, Djohana Laurent, Pauline Peyrouze, Amine Belhabri, Yann Guillermin, Frederic Mazurier, Meyling Cheok, Marie-Charlotte Audry-Deschamps, Magalie Faivre, Véronique Maguer-Satta, Sylvain Lefort

**Affiliations:** 1 CNRS UMR5286, Centre de Recherche en Cancérologie de Lyon, 69008 Lyon, France; 2 Inserm U1052, Centre de Recherche en Cancérologie de Lyon, 69008 Lyon, France; 3 Universite Claude Bernard Lyon 1, CRCL, 69000 Lyon, France; 4 Univ Lyon, UCBL, INSA Lyon, ECL, CNRS, CPE Lyon, INL, UMR5270, 69622 Villeurbanne, France; 5 Centre Léon Bérard, 69000 Lyon, France; 6 CNRS-UMR9020, INSERM-U1366, University of Lille, Lille Hospital, CRC-Lille Cancer Research Center of Lille, Lille, France; 7 Univ. Rennes, CNRS, Inserm, IGDR (Institut de Génétique et Développement de Rennes), UMR6290, ERL U1305, 35000 Rennes, France

**Keywords:** Acute myeloid leukaemia, Cancer therapeutic resistance

## Abstract

Despite extensive research and intensive use of chemotherapies in clinics, the 5-year overall survival of acute myeloid leukemia (AML) patients does not exceed 20%. The clonal expansion of leukemic blasts leads to modifications of the bone marrow physical properties, including increased extracellular matrix stiffening, upregulation of intramedullary pressure and reduction of the space available for cells. These biomechanical modifications are speculated to alter therapeutic response and cause treatment resistance. To address this, we herein focused on the role of mechanotransduction pathways in AML. Analysis of primary AML samples or cell lines revealed that BMPR1B and TAZ/TEAD but not YAP levels were higher after patient relapse or in cells resistant to cytarabine or venetoclax. In addition, highly confined resident mesenchymal stem cells expressed higher levels of BMP4, which in turn specifically activated AML-resistant cells. In these cells, TAZ expression was associated with improved adhesion to microenvironmental components and increased intrinsic deformability. Finally, using a 3D human bone marrow-like model, we showed that targeting BMPR1B or TAZ/TEAD in combination with cytarabine impaired persistence of AML primary cells within the AML niche. Future therapeutic approaches could involve BMPR1B and/or TAZ/TEAD targeting in the context of AML patients refractory to chemotherapy or after relapse.

## Introduction

Hematopoiesis occurs in the bone marrow (BM) where hematopoietic stem cells (HSCs) self-renew or differentiate into myeloid or lymphoid blood lineages. HSC properties are tightly regulated by their direct cell to cell interaction with the BM microenvironment (or niche), which is composed of several cell types including adipocytes, osteoblasts, endothelial cells and mesenchymal stem cells (MSCs) [1], and by acellular components such as cytokines and extracellular matrix (ECM) proteins, mainly fibronectin or collagens. Indeed, ECM- embedded cytokines, such as TGFβ can trigger HSC adhesion and quiescence [2, 3], while substrate stiffness cues were shown to regulate their differentiation ability [4].

Acute myeloid leukemia (AML) is a highly heterogeneous disease characterized by a broad spectrum of molecular alterations, resulting in the hyperproliferation of immature myeloblasts due to a differentiation blockage. The number of recurrent cytogenetic abnormalities and mutations in AML influence disease phenotype, response to conventional therapies, risk of relapse, and survival [5]. Intensive induction regimen of combined cytarabine and anthracycline remains the standard-of-care for most AML patients. However, complete remission with this approach does not exceed 15% in patients over 60, who often display an increased risk of treatment-related mortality and primary or secondary resistance to chemotherapies [5]. Despite the recent success of a new therapeutic combination of venetoclax and hypomethylating agents, resistance mechanisms have already been reported, prompting research into novel therapeutic targets.

In AML, the rapid expansion of myeloblasts alters the physical properties (through rapid lack of space) and cell populations of the BM niche. Indeed, various studies demonstrated a bias in MSC differentiation in AML either towards an osteoblastic or adipocytic fate [6, 7]. Moreover, some level of BM fibrosis, illustrated by significant collagen or reticulin deposit, is present in a third of leukemia patients at diagnosis [8]. In addition to ECM substrate stiffening, such as in fibrosis, the overall intramedullary BM pressure is also higher in AML patients [9]. Finally, cytokines secreted by the BM niche such as CXCL12 (also known as SDF-1) were shown to contribute to AML progression and chemoresistance, and their targeting re-sensitized AML cells to treatment [10, 11]. In chronic myeloid leukemia, cytokines are linked with quiescence [12] and chemoresistance through BMP4-BMPR1B interactions [13]. In AML, BMP4 is responsible for the acquisition of stem-like features [14], and anti-apoptotic properties through the expression of the *mixl1* gene [15].

Interestingly, BMP signaling can be activated by ligand/receptor interactions, but also by mechanical stimuli [16]. Proteins of this pathway orchestrate bone and cartilage formation, and tissue organization throughout the body, and were shown to crosstalk with integrins [16]. These receptors are responsible for sensing the environment, and can form focal adhesions, linking the actin cytoskeleton to the ECM through adaptor proteins and by activating FAK (focal adhesion kinase) through phosphorylation. BMP signaling and integrins control cell proliferation [17], or differentiation [18]. These pathways also crosstalk with one of the major mechanotransduction pathways in adherent cells, namely YAP/TAZ [19, 20]. YAP/TAZ proteins, generally inhibited by the Hippo pathway, are freed with cell stretching and adhesion to a stiff matrix [21], and enter the nucleus, where they bind to TEAD transcription factors, and further activate the transcription of target genes (including *cyr61, ctgf*). YAP/TAZ regulate cell migration, proliferation, differentiation and self-renewal, and are frequently dysregulated in solid cancers [22]. Moreover, some highly aggressive cancers are deficient in YAP (YAP^off^), including leukemias [23], and its re-expression was shown to induce cell cytostasis, with expression of integrins and ECM [23]. Though it was reported that YAP/TAZ forced expression in multiple myelomas leads to cell death [24, 25], while their inhibition causes apoptosis in promyelocytic AML [26], their role is poorly described in AML, particularly in chemoresistant leukemia cells.

Given that AML have a mechanically-dysregulated microenvironment, we wondered whether mechanotransduction pathways could modify AML chemoresistance. Using AML cell lines and primary patient samples after relapse, we investigated the role of both BMP and YAP/TAZ pathways in AML chemoresistance. We show that the mechanical properties (adhesion to various stroma, and intrinsic stiffness) and mechanotransduction pathways are altered in AML cells. In addition, we identified that TAZ expression is central for the control of mechanical properties. Finally, we validated that BMP or TAZ/TEAD targeting impairs chemoresistant AML cell growth in a human 3D microphysiological bone marrow model.

## Methods

### Cells

Bone marrow samples were obtained either from healthy BM donors for allogeneic transplant or from AML patients at diagnosis or relapse, after receiving chemotherapy. All donors provided written informed consent in accordance with the Declaration of Helsinki. Studies were approved by local ethics committee bylaws (Agreement from Centre Léon Bérard Biological Resources Center). Mesenchymal stem cells (MSCs) were isolated from BM samples prior to Ficoll gradient separation and cultured, whereas MonoNuclear Cells (MNCs) were isolated after Ficoll gradient separation only.

The AML cell lines used, ML2 (RRID:CVCL_1418) and OCI-AML3 (RRID:CVCL_1844) were cultured in RPMI 1640 with 10% fetal bovine serum (FBS). Resistant cells were permanently cultured with cytarabine (Ara-C) or venetoclax at 1 µM. ML2-sensitive (ML2-S) and ML2-resistant (ML2-R) cell lines, as well as OCI-AML3-S and OCI-AML3-R, were cultured in RPMI 1640 10% FBS media, as previously described [27]. Stable ML2-R and OCI-AML3-R cell lines harboring shCtl or dual shYAP/TAZ referred to as shTAZ#1 and shTAZ#2 (constructs generously given by Rob Bremner lab) have been made. The HS-27A (RRID:CVCL_3719) and HMEC-1 (RRID:CVCL_0307) cell lines were obtained from ATCC and cultured in RPMI 1640 containing 10% FBS or MCBD131 containing 10% FBS, 1% glutamax, hydrocortisone at 1 µg/mL and EGF at 10 ng/mL. Cells were treated with BMP4 at 10 ng/mL (R&D System, 314-BP), E6201 at 100 nM (Strategia), or verteporfin at 0.25 µM (Sigma, (RRID:SCR_000488) SML0534) alone or in combination with Ara-C at 1 µM.

For cells cultured in the 3D bone-marrow model, we followed previously described protocols [28], using BMP4-overexpressing HS-27A and -HMEC-1 for 3 weeks. When human 3D bone marrow systems were ready, 500,000 primary relapsed AML MNCs were introduced for a day, prior to 2 cycles of Ara-C chemotherapy (4 days of treatment followed by 3 days without treatment), over 2 weeks.

### Immunofluorescence staining

Cells were plated onto glass coverslips coated with fibronectin (Sigma, F0895-2MG) at 40 µg/mL diluted in 0.1 M NaHCO3, incubated for 30 min at 37 °C, then washed with PBS before seeding the cells at 0.2 M/mL. Alternatively, cells were also plated with stromal cells seeded a day prior to the experiment. For stiff/soft matrices, cells were plated on hydrogels (0.5 kPa or 4 kPa Softwell -Matrigen) coated with fibronectin. After 24 h, cells were gently washed with PBS then fixed for 20 min in paraformaldehyde (PFA) 4% at room temperature. They were washed 3 times with PBS before incubation for 30 min at room temperature with blocking and permeabilizing solution (BSA 3%+ Triton 0.3% +PBS 1X). Coverslips were then incubated with primary antibody, diluted in 1% BSA-PBS, overnight at 4 °C. The next day, coverslips were washed 3 times with PBS before incubation of secondary antibodies and nuclear staining, diluted in 3% BSA-PBS, for 45 min. Finally, after 4 washes, the coverslips were dried and mounted in DAKO mounting medium. Adherent cells were imaged using a x10 (air) optical microscope (Zeiss Primovert). Adherent cells were counted on 4 different fields/condition, using the particle analysis function on ImageJ 1.54p. Colocalization analysis was analyzed using ImageJ Coloc2 Plugin quantifying Pearson’s coefficient in z-stack acquired fluorescent signal using x63 (oil) magnification confocal microscope Leica Stellaris 5.

Primary antibodies used were anti-Pan-TEADs (Cell Signaling Technology, #13295) at 1:600, anti-BMPR1B (Santa Cruz, sc-293428) at 1:300, Phalloidin-rhodamine (Life, R415) at 1:800, Anti-P-FAK Tyr397 (Thermofisher, 700255) at 1:400 and WWTR1/TAZ (Proteintech, 66500-1-Ig) at 1 :200. We then added secondary antibodies: Goat anti-mouse Alexa Fluor 405 (Invitrogen, A31553) at 1:1,000, Goat anti-mouse IgG Dylight 488 (Thermo scientific, 35502), Goat anti-Rabbit IgG Dylight 550 (Thermo scientific, 84540) at 1 :1,000, Hoescht 33342 (Thermo scientific, H3570) at 1 :10,000 or Sytox Deep Red (Thermofisher, S11380) at 1:30,000 to stain the nucleus.

### Western blotting

Cells were harvested, pelleted, and lysed with RIPA buffer. Proteins were then quantified by spectrophotometry using a BSA standard curve, and 30 µg were loaded into a 10% acrylamide gel. Polyvinylidene fluoride (PVDF) membranes previously incubated in ethanol were used for dry transfer with Transblot Turbo (BioRad). Membranes were then incubated for 1 h with 5% BSA or milk at room temperature for saturation. The primary antibodies were incubated at 4 °C overnight. These were: anti-phospho-Erk (Cell Signaling Technology, #4370) at 1:8000, anti-phospho-Smad1/5/8 (Cell Signaling Technology, #13820) 1:500, anti-BMPR1b (Abcam, ab78417) at 1:500, anti-phospho-P38 (BD Biosciences, #612288) at 1:1000, anti-P38 (BD Biosciences, #612168) at 1:1,000, anti-GAPDH (Cell Signaling Technology, #8884) at 1:10,000 dilution in 5% BSA. Also, anti-Erk (Cell Signaling Technology, #4695), anti-Smad1/5/8 (Santa Cruz, sc-6031-R) at 1:1,000, anti-TAZ (Cell Signaling Technology, #72804) at 1:500, anti-Pan-TEADs (Cell Signaling Technology, #13295) at 1:1,000 dilution in 5% dry milk. After Tris-buffered saline-Tween (TBST) washes, membranes were incubated at room temperature for 1 h with horseradish peroxidase (HRP) conjugated with goat anti-rabbit or goat anti-mouse secondary antibodies at 1:10,000 in 1% milk. Samples were then washed with TBST before electrochemiluminescence (ECL) visualization on Chemidoc (BioRad). Proteins were quantified using ImageLab software.

### Flow cytometry

Cells were stained with antibodies tagged with fluorescent probes and their counterpart IgG for 20 min at 4 °C in the dark. Cells were then washed with PBS. The antibodies used were supplied by BD Biosciences (RRID:SCR_013311): conjugated BV500 CD45 (#560777), conjugated APC-H7 CD45RA (#560674), conjugated BV605 CD90 (#747750), conjugated APC CD34 (#555824), conjugated PeCy7 CD38 (#335825), conjugated BV711 CD123 (#751833), conjugated PE CD47 (#556046), conjugated PECy5 CD29 (#559882).

For apoptosis assays, cells were washed with PBS then binding buffer. An annexinV stain (BD Bioscience, 556547) was added to the cells and incubated for 15 min at room temperature in the dark. After 2 washes with binding buffer, cells were kept on ice. Propidium iodide (PI) was added 5–15 min before flow cytometry analyses on LSRFortessa (BD) at the CRCL Flow Cytometry core facility.

### Statistical analyses

Mean comparisons were performed with a bilateral Mann-Whitney or t-test (paired or unpaired as required), using GraphPad Prism software ((RRID:SCR_002798), La Jolla, CA). Significant *P*-values are indicated by asterisks in the Figures. *P ≤ 0.05, **P ≤ 0.01, ***P ≤ 0.001 and ****P ≤ 0.0001 were considered statistically significant.

## Results

### Induction of specific mechanotransduction pathways in relapsed AML patients

**Fig. 1:**
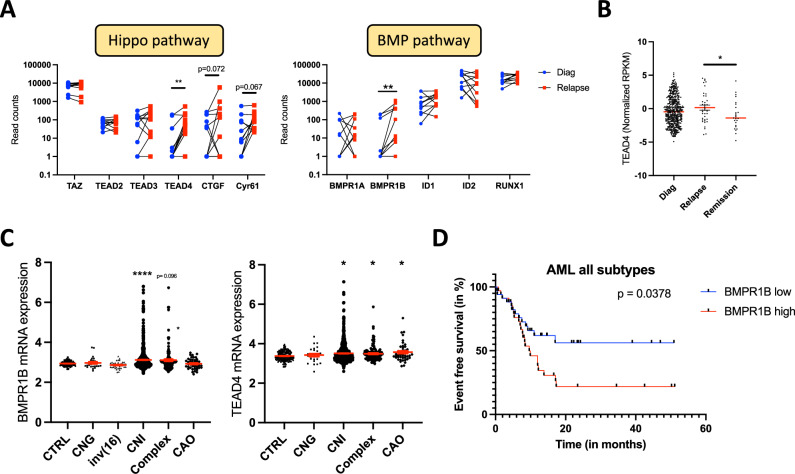
Relapsed AML patients display increased BMP and Hippo elements. **A** Relative mRNA expression from paired AML primary samples at diagnosis and after relapse (GSE106291), of elements from the Hippo pathway (left panel; TAZ, TEAD2, TEAD3, TEAD4, CTGF, Cyr61), and BMP pathway (right panel; BMPR1A, BMPR1B, ID1, ID2, RUNX1). Values are shown as RPKMs and represent 11 patients for each gene. **B** mRNA expression of TEAD4 from AML samples at diagnosis (*n* = 450), relapse (*n* = 37) or remission (*n* = 25) stages (OSHU dataset). Values are shown as normalized RPKMs. **C** BMPR1B (left panel) and TEAD4 (right panel) mRNA expression according to AML cytogenetic status (GSE147515). CNG cytologically-normal with good prognosis, CNI cytologically-normal with intermediate prognosis, CAO cytologically-abnormal not-otherwise specified. Values are shown as normalized RPKMs. **D** Kaplan-Meier curves of event-free survival from GSE147515, with respect to BMPR1B mRNA level in all AML subtypes (*n* = 68).

Since the mechanical properties of the AML niche are dysregulated compared to normal BM, we wondered whether reciprocal mechanical modulation occurred in leukemic cells. To address this, we first analyzed if any mechanotransduction pathway was dysregulated in AML cells. Using a first dataset of paired primary AML samples at diagnosis and after relapse, we identified no change in the expression of known mechanosensors, such as ion channels (Piezo, TRPV1, TRPV2) or integrins (Supplementary Fig. 1A). However, in relapsed AML patients, the YAP/TAZ partner and DNA-binding protein TEAD4, as well as BMPR1B (Fig. 1A, left and right panels), were overexpressed. Using a second dataset, we confirmed that both TEAD4 and BMPR1B (Fig. 1B and Supplementary Fig. 1B–D) were increased in relapse AML patients compared to AML patients in remission, suggesting that YAP/TAZ signaling and BMPR1B receptor may play a role in chemoresistance. Interestingly, neither YAP/TAZ nor BMP elements were overexpressed in AML patients at diagnosis compared to normal BM (NBM) (Supplementary Fig. 1E). This is consistent with the observation that leukemias are YAP/TAZ^off^ cancers [23], but suggests that some mechanotransduction pathways are re-activated when patients become resistant to treatment. Moreover, using horizontal meta-analysis of integrated transcriptomic data of AML patients, we observed that BMPR1B and TEAD4 were overexpressed in CNI and complex karyotypes compared to control NBM (Fig. 1C). Lastly, analysis of the event-free survival of AML patients with all subtypes, showed that those with high BMPR1B expression (Fig. 1D, Supplementary Fig. 1F), but not TEAD4 (Supplementary Fig. 1G), had a worse prognosis. Thus, while elements of BMPR1B and Hippo signaling may be involved in chemoresistance, BMPR1B alone may constitute a prognostic factor at diagnosis.

### TEAD and BMP4/BMPR1B are overexpressed and active in AML chemoresistant models

**Fig. 2:**
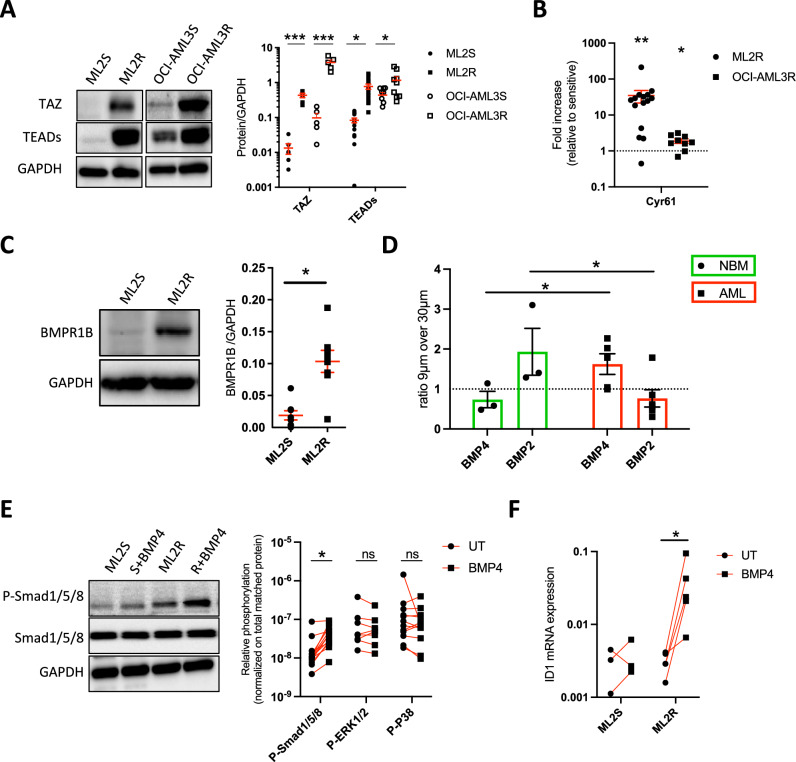
TEAD and BMP4/BMPR1B are overexpressed and active in AML chemoresistant models. **A** Western blots showing TAZ and TEADs levels (relative to GAPDH) of AraC-sensitive (S) or Ara-C-resistant (R) ML2 or OCI-AML3 cells grown for 24 h. Individual data are from independent experiments. **B** CYR61 mRNA expression represented as fold change from Ara-C-resistant ML2 (*n* = 15) and OCI-AML3 (*n* = 9) cells normalized against Ara-C-sensitive cells ± SEM. **C** Western blots showing BMPR1B levels (relative to GAPDH) of Ara-C-sensitive (S) or Ara-C-resistant (R) ML2 cells grown for 24 h. Individual data are from independent experiments. **D** BMP2 and BMP4 mRNA expression in primary mesenchymal stem cells (MSCs) from normal bone marrow (NBM) (*n* = 3) or AML at diagnosis (*n* = 4). BM MSCs were grown under 9 μm confinement for 72 h and normalized against cells confined at 30 μm. **E** Western blots showing P-Smad1/5/8, P-Smad1/5/8 (relative to GAPDH) of Ara-C-sensitive (S) or Ara-C-resistant (R) ML2 cells treated or not with BMP4 for 30 min. Individual data are from independent experiments. Dot plot showing phosphorylation levels (normalized on total levels) in Ara-C-resistant ML2 cells +/- BMP4. Individual data are from independent experiments. **F** ID1 mRNA expression of Ara-C-sensitive (S) or Ara-C-resistant (R) ML2 cells treated or not with BMP4 for 48 h. Individual data are from independent experiments.

To determine if the mechanical properties of cells are associated with AML chemoresistance, we first used 2 different AML cell lines (ML2 or OCI-AML3), that were both rendered resistant (-R) to 1 μM cytarabine (AraC) treatment, which we compared to their sensitive (-S) parental cell lines (Supplementary Fig. 2A). To confirm the importance of mechanotransduction pathways in the resistance of cells to chemotherapy, we first validated that TAZ and TEAD were more abundant in chemoresistant cells compared to AraC-sensitive cells (Fig. 2A). In addition, Cyr61 mRNA was also overexpressed by 3-30-fold in both ML2 and OCI-AML3 AraC-resistant (ML2R and OCI-AML3R) cells compared to AML-sensitive cell lines (Fig. 2B), reflecting the efficacy of TAZ/TEAD activation. On the other hand, no alteration in YAP levels was observed using flow cytometry (Supplementary Fig. 2B) or Western blotting (Supplementary Fig. 2C). We then confirmed that BMPR1B levels were higher in ML2R (Fig. 2C) and OCI-AML3R cells (Supplementary Fig. 2D). To identify the signaling pathways associated with chemoresistance, we tested basal activity of some major BMP-related pathways and observed a significant reduction in P-ERK1/2, increase in P-P38 but not significant difference in P-Smad1/5/8 in ML2R cells compared to ML2S cells (Supplementary Fig. 2E).

AML progression profoundly remodels the bone marrow niche, increasing cellular density and stiffness, which imposes mechanical constraints on stromal and endothelial cells. Such compressive stress is known to activate mechanotransduction pathways, including BMP signaling [29], and to alter the secretion profile of niche-derived factors. Given that BMP have been reported to respond to mechanical cues in various tissues [16] and BMP4 accumulate in AML BM plasma [14], we used a previously developed confinement system [30] to investigate niche cell response. Primary BM MSCs from healthy donors or from AML patients at diagnosis, or human endothelial HMEC-1 cells, were cultured under a confined space of 9 µm for 3 days before harvesting their mRNA (Supplementary Fig. 2F). Cell confinement induced an increase in BMP4 mRNA in AML MSCs and endothelial cells (Fig. 2D and Supplementary Fig. 2G), whereas BMP2 was upregulated in NBM MSCs (Fig. 2D). We hypothesized that BMP4 release from cells grown in a confined niche cells could thus act as a mechanosensitive signal that reinforces BMP-Smad pathway in AML cells, contributing to survival and chemoresistance. We then applied BMP4 treatment for 30 min (Fig. 2E) or 48 h (Fig. 2F) in both sensitive and chemoresistant ML2 cells. While BMP4 treatment of chemoresistant ML2 cells led to a specific increase in Smad1/5/8 phosphorylation (Fig. 2E) and activation of the BMP target gene *ID1* (Fig. 2F), it had no effect on P-Smad1/5/8, P-ERK1/2 or P-38 (Supplementary Fig. 2H) or ID1 mRNA expression (Fig. 2F) in AraC-sensitive ML2 cells. Overall, our results suggest that compressive stress of AML MSCs is associated with increased BMP4 accumulation leading to the induction of BMP signaling only in resistant AML cells, as well as increased activation of TAZ/TEAD activity.

### Increased cell deformability promotes adhesion of chemoresistant AML cells to softer substrates

**Fig. 3:**
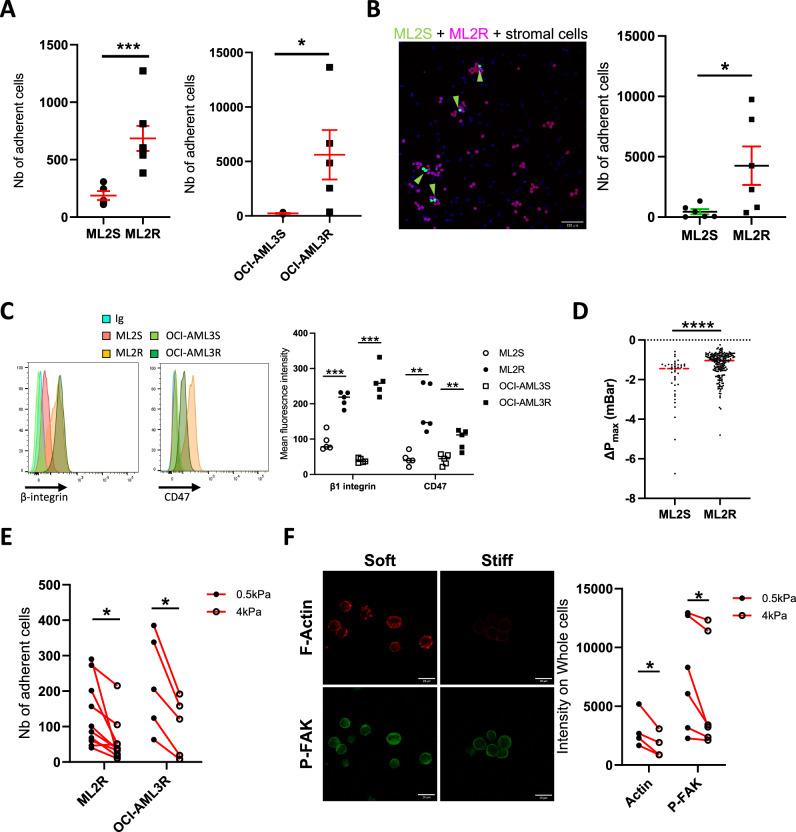
Increased cell deformability promotes adhesion of chemoresistant AML cells to softer substrates. **A** Number of adherent cells after 24 h culture on a fibronectin-coated layer for ML2 (left panel) or OCI-AML3 (right panel) cells. Individual data are from independent experiments. **B** Representative images of a 24 h co-culture of HS-27A stromal cells with ML2S (green) and ML2R (magenta). Scale bar, 100 μm. Dot plot showing number of adherent leukemic cells from independent experiments. **C** Staining intensity of β-integrin (left panel) or CD47 (right panel) expression in ML2 and OCI-AML3 cells. Dot plot showing Mean Fluorescent Intensity (MFI) of β-integrin and CD47 in Ara-C-sensitive (open circles/squares) or Ara-C-resistant (solid circles/squares) cells. Individual data are from independent experiments. **D** Dot plot representing the maximum pressure drop of ML2S, associated with greater stiffness, compared to ML2R. Individual dots represent a single value per cell (from 4 independent experiments). **E** Number of adherent ML2R or OCI-AML3R cells on soft (0.5 kPa) or stiff (4 kPa) fibronectin coated hydrogels for 24 h. Individual data are from independent experiments. **F** Representative micrographs of F-actin and P-FAK staining from Ara-C-resistant ML2 cells coated on soft (0.5 kPa) or stiff (4 kPa) hydrogels. Scale bar, 20 μm. Dot plot showing F-actin and P-FAK intensity from ML2R cells seeded onto soft or stiff hydrogels. Individual data are from independent experiments.

Since cell adhesion to the BM microenvironment has been reported to enhance initial treatment response in AML [31], we first evaluated if established resistant AML cells displayed different adhesion patterns, on fibronectin-coated plates (Fig. 3A), or on MSC model HS-27A (Fig. 3B). On both substrates, AML-resistant cells were more adherent than AML-sensitive cells (Fig. 3A, B), with a 3-20-fold increase in cell adhesion. We next investigated if the increased expression of adhesion molecules was associated with chemoresistance. Both ML2 and OCI-AML3 cells overexpressed β1 integrins and CD47 in AML-chemoresistant cells (Fig. 3C), but not α5 integrin (Supplementary Fig. 3A). CD123 was only overexpressed in OCI-AML3-chemoresistant cells (Supplementary Fig. 3A). In solid cancers, greater cell deformability is associated with increased expression of mechanoreceptors, as well as enhanced invasive and metastatic processes [32]. To ascertain whether intrinsic cell stiffness was altered in chemoresistant AML cells, we used an innovative microfluidic strategy – referred to as the pressure drop measurement – to explore hydrodynamic resistance associated with the flow of a cell in a microfluidic constriction [33] (Supplementary Fig. 3B). In this setting, the greater the drop in pressure associated with the flow of a cell in the constricted device, the stiffer the cell is for a given volume. We observed that ML2R cells displayed decreased intrinsic stiffness compared to ML2S cells (Fig. 3D), which was further confirmed using Atomic Force Microscopy (Supplementary Fig. 3C). Nevertheless, this difference in deformability was not observed in OCI-AML3 cells (Supplementary Fig. 3D), possibly due to a weaker fold increase in TEAD compared to ML2 cells (Fig. 2A). Finally, since chemoresistant AML cells were more deformable, we wondered whether their cell adhesion capacity would be sensitive to changes in microenvironmental stiffness, using Soft (0.5 kPa) or Stiff (4 kPa) fibronectin-coated hydrogels, mimicking mechanical properties close to those found in the bone marrow, ranging from the sinusoidal region to the endosteal region, respectively [34]. After 24 h, analysis of adherent cells using the Sytox nuclear dye revealed that chemoresistant AML cells adhered better to soft substrates compared to stiff substrates (Fig. 3E), as opposed to both HS-27A and MCF10A cells (Supplementary Fig. 3E). This decrease in adhesion to stiffer hydrogels was also associated with a lack of actin cytoskeleton organization and a reduced P-FAK activation (Fig. 3F). Thus, our results suggest that increased deformability of AML-chemoresistant cells is associated with enhanced adhesion to softer microenvironments.

### Venetoclax-resistant AML cells also display high levels of BMPR1B and TEAD, associated with enhanced adhesion and increased deformability

**Fig. 4:**
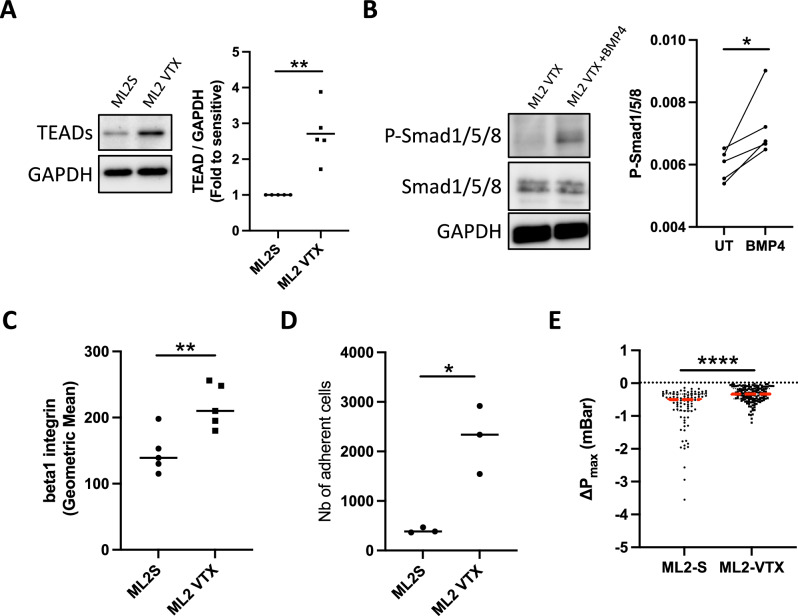
Induction of mechanotransduction pathways, adhesion and cell deformability in venetoclax-resistant cells. **A** Western blots showing TEADs levels (relative to GAPDH) of venetoclax-sensitive (S) or -resistant (VTX) ML2 cells grown for 24 h. Individual data are from independent experiments. **B** Western blots showing P-Smad1/5/8, P-Smad1/5/8 (relative to GAPDH) of venetoclax-resistant (VTX) ML2 cells treated or not with BMP4 for 30 min. Individual data are from independent experiments. **C** Dot plot showing the mean fluorescence intensity (MFI) of β-integrin in venetoclax-sensitive (circles) or -resistant (squares) cells. Individual data are from independent experiments. **D** Dot plot showing number of adherent leukemic cells (CD45 + ) retrieved from co-culture on HS-27A cell layer after 24 h. Individual data are from independent experiments. **E** Dot plot representing the maximum pressure drop of ML2S, associated with greater stiffness, compared to ML2 VTX. Individual dot represent a single value per cell (from 4 independent experiments).

Given the drastic increase in AML patients displaying resistance to chemotherapy, alternative therapies such as venetoclax were developed, albeit they also rapidly led to drug resistance in numerous patients [35]. Here, we developed another model of ML2 cells resistant to venetoclax, ML2-VTX, since AraC-sensitive cells also responded to this treatment (Supplementary Fig. 4A). ML2-VTX presented elevated BMPR1B and TEAD protein levels but not TAZ (Fig. 4A, Supplementary Fig. 4B) and increased expression of TAZ/TEAD target genes, such as *Cyr61* or *CTGF* (Supplementary Fig. 4C). In addition, ML2-VTX cells treated with BMP4 had greater levels of P-Smad1/5/8 (Fig. 4B). As for chemoresistant AML cells, ML2-VTX also displayed increased β1 integrin expression (Fig. 4C), adhesion to HS-27A stromal cells (Fig. 4D and Supplementary Fig. 4D) and increased cell deformability (Fig. 4E), compared to sensitive ML2 cells. Collectively, our results suggest that activation of mechanotransduction pathways is not specific to cells resistant to cytarabine but may develop in other contexts of resistance to chemotherapy, such as for venetoclax.

### Targeting the Hippo pathway leads to alterations in the biomechanical properties of chemoresistant AML cells

**Fig. 5:**
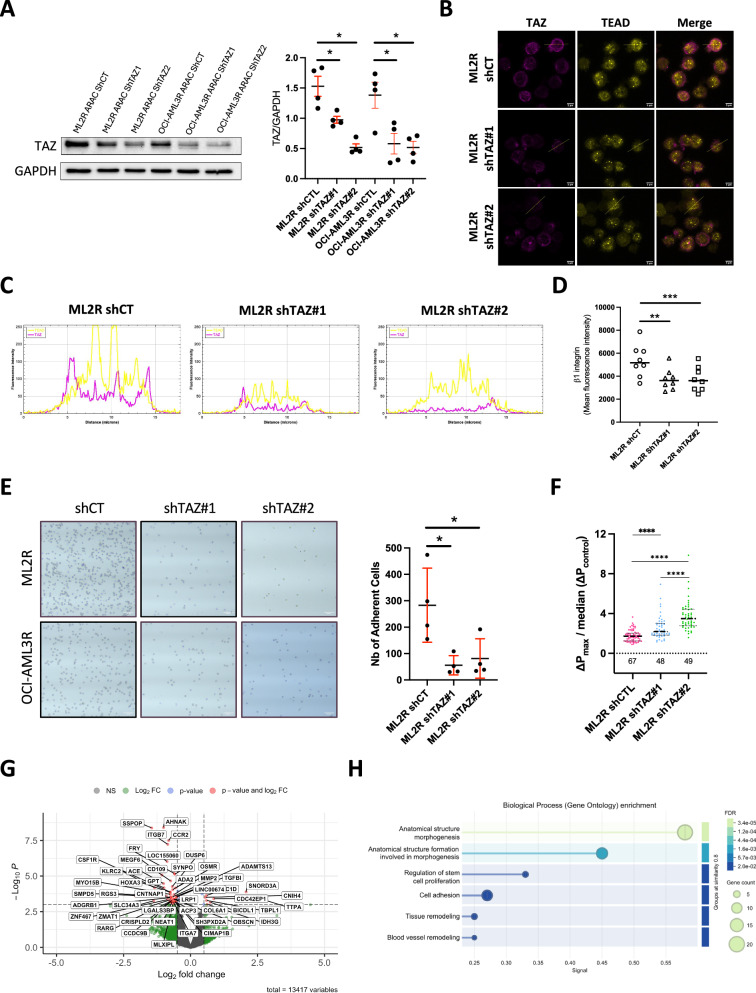
TAZ expression is directly correlated to cell deformability and adhesion. **A** Western blots showing TAZ levels (relative to GAPDH) of Ara-C-resistant (R) ML2 or OCI-AML3 cells harboring shCtl, shTAZ#1 or shTAZ#2. Individual data are from independent experiments. **B** Representative images of TAZ and TEAD staining from Ara-C-resistant ML2 cells harboring shCtl, shTAZ#1 or shTAZ#2. Scale bar, 5 μm. **C** Graph showing Line profile colocalization analysis from ML2R cells harboring shCtl, shTAZ#1 or shTAZ#2. **D** Dot plot showing MFI of β-integrin in Ara-C-resistant (R) ML2 cells harboring shCtl, shTAZ#1 or shTAZ#2. Individual data are from independent experiments. **E** Representative images of 24 h culture on a fibronectin-coated layer of ML2R cells harboring shCtl, shTAZ#1 or shTAZ#2. Scale bar, 100 μm. Dot plot showing the number of adherent cells for ML2R cells harboring shCtl, shTAZ#1 or shTAZ#2. Individual data are from independent experiments. **F** Dot plot representing the maximum pressure drop of ML2R cells harboring shCtl, shTAZ#1 or shTAZ#2. Individual dot represent a single value per cell (from 3 independent experiments). **G** Volcano plot representation of the differentially expressed genes (DEGs) in ML2R cells harboring shCtl or shTAZ#2. DEGs are pseudocolored in green when not significantly different or in red when a significant difference is measured. **H** Gene ontology analysis performed on transcriptomic data obtained from ML2R cells harboring shCtl or shTAZ#2. The graph shows the 6 most represented GO terms among differentially downregulated DEGs compared to shCtl. The data were obtained from 3 biological replicates.

Since chemoresistant AML cells displayed increased expression of both TAZ/TEAD, associated with increased cell adhesion and deformability, we next wondered if there was a direct correlation between TAZ expression level and the biomechanical functions of AML cells. We used 2 different shRNA against TAZ that inhibited TAZ by 30 to 60% in both ML2R and OCI-AML3R (Fig. 5A), without affecting the expression of TEAD, BMPR1B or Smad1/5/8 phosphorylation (Supplementary Fig. 5A). First, even if some remaining TAZ was available, we observed a reduced co-localization of TAZ with TEAD in the nucleus of ML2R cells harboring shTAZ (Fig. 5B, C, Supplementary Fig. 5B). In addition, we noticed a decreased expression of β1-integrin in shTAZ ML2R and OCI-AML3R (Fig. 5D and Supplementary Fig. 5C, D). In the context of cell adhesion to fibronectin, TAZ inhibition was also associated with a reduction of AML-resistant cells bound to ECM (Fig. 5E and Supplementary Fig. 5E). Moreover, we observed in ML2R and OCI-AML3R harboring shTAZ, particularly with shTAZ#2, an increased stiffness compared to shCTL AML-resistant cells (Fig. 5F and Supplementary Fig. 5F). Finally, transcriptomic analyses of ML2R shCtl and shTAZ cells confirmed that TAZ inhibition was associated with a decrease in signatures of structural cell organization and cell adhesion (Fig. 5G, H and Supplementary Fig. 6A, B). All together, our results showed that the level of TAZ expression in chemoresistant AML cells was correlated to cell-matrix adhesive properties and regulates intrinsic cell stiffness.

### Targeting BMPR1B or TAZ/TEAD interactions impairs AML bone marrow persistence

**Fig. 6:**
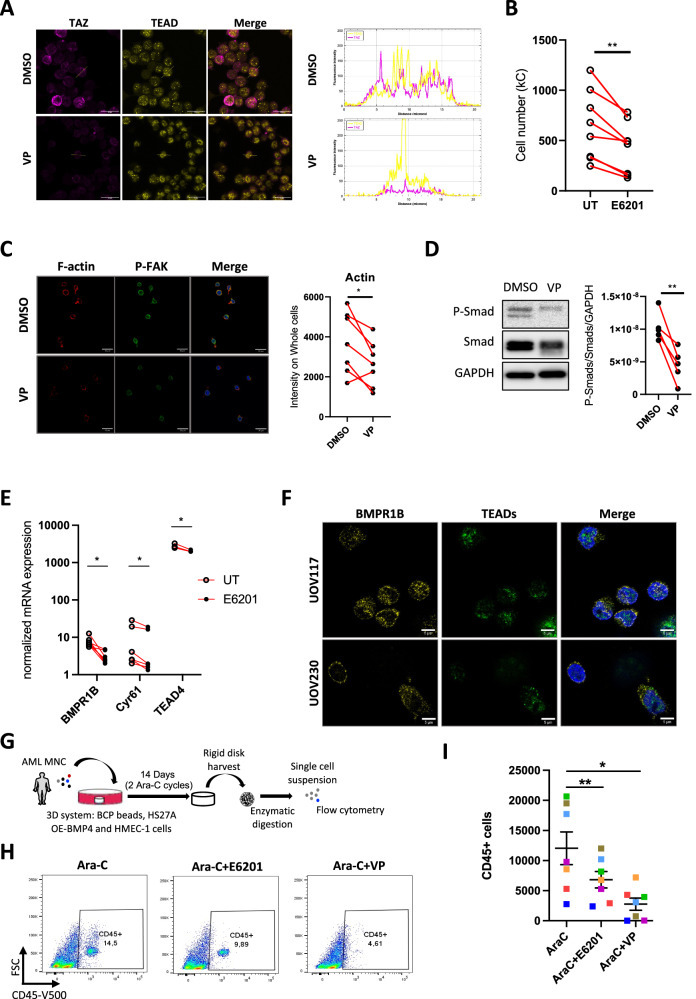
Targeting BMPR1B or TAZ/TEAD interactions impair AML bone marrow persistence. **A** Representative images of TAZ and TEAD staining from Ara-C-resistant ML2 cells treated with DMSO or verterporfin (VP) for 24 h. Scale bar, 30 μm. Graph showing Line profile colocalization analysis from DMSO- or VP-treated ML2R cells. **B** Number of ML2R cells after 72 h culture, treated or not with E6201 (100 nM). **C** Representative images of F-actin and P-FAK staining from Ara-C-resistant ML2 cells treated with DMSO or verterporfin (VP) for 24 h. Scale bar, 30 μm. Dot plot showing F-actin intensity from DMSO- or VP-treated ML2R cells. **D** Western blots showing P-Smad1/5/8, P-Smad1/5/8 (relative to GAPDH) from Ara-C-resistant ML2 cells treated with DMSO or VP for 24 h. Individual data are from independent experiments. **E** BMPR1B, CYR61 and TEAD4 mRNA expression from Ara-C-resistant ML2 cells untreated (UT) or treated with E6201 for 48 h. **F** Representative images of BMPR1B and TEADs staining of primary samples from relapsed AML patients. Scale bar, 5 μm. **G** Schematic diagram of the experimental set-up of relapsed primary AML samples cultured on the 3D human bone marrow model, with a combination of Ara-C and E6201 or VP for 2 weeks. **H** Representative graphs of CD45+ cells retrieved from dissociated 3D human bone marrow models, after 2 weeks of combined treatment. (**I**) Dot plot showing number of CD45+ cells. Each colored dot represents different relapsed primary AML patients.

Considering the activation of both BMRP1B and TAZ/TEAD elements in AML resistant cells, we next wondered whether targeting these pathways, using verteporfin (a drug preventing the interaction of TEADs with YAP/TAZ) or E6201 (BMPR1B inhibitor), would impact their survival. First, we confirmed that verteporfin, but not E6201, impaired TAZ and TEAD colocalization in ML2R cells (Fig. 6A and Supplementary Fig. 7A). We then observed that verteporfin, alone or in combination with AraC, promoted apoptosis (Supplementary Fig. 7B), while E6201 had a more pronounced cytostatic effect (Fig. 6B, Supplementary Fig. 7C), suggesting that verteporfin and E6201 could have complementary effects on both cell survival and cell proliferation. In addition, staining for F-actin was weaker when chemoresistant AML cells were incubated with verteprofin (Fig. 6C), but not E6201 (Supplementary Fig. 7D). Together, our results suggest that E6201 could act on cell proliferation, while verteporfin seems to change cytoskeletal properties. We then considered a potential crosstalk between the BMP and Hippo pathways. First, we observed that ML2R cells displayed reduced Smad1/5/8 phosphorylation levels when incubated with verterporfin (Fig. 6D), and that E6201 reduced BMPR1B expression and impaired both Cyr61 and TEAD4 mRNA levels (Fig. 6E), suggesting that BMP and Hippo pathways could be interconnected.

Since BMPR1B and TAZ/TEAD inhibitors had significant effects on either cell proliferation or cell survival, we assessed their impact on AML-resistant (refractory or relapse) primary patient samples. These were obtained from patients of various ages, genders, genetic landscapes and leukemic stem fractions (CD34 + CD38-CD45RA + ) fractions (Supplementary Fig. 8A, B). AML cells were maintained in 2D co-culture with engineered BMP4-overexpressing HS-27A cells for 1 or 3 days, in combination with AraC/E6201/verteporfin, before immunofluorescence or flow cytometry (Supplementary Fig. 8C). AML samples from chemoresistant patients were positive for both BMPR1B and TEADs (Fig. 6F), consistent with their potential involvement in resistance of primary tumors to chemotherapy. After harvesting CD45+ leukemic fractions from 3 day co-cultures, we confirmed that AraC had no effect on cell number (Supplementary Fig. 8D, E), nor did its combination with verteporfin, although its combination with E6201 slightly decreased AML cell proliferation (Supplementary Fig. 8E). Given that the AML BM niche is more complex than 2D co-culture experiments with stromal cells, we tested longer exposure periods of various drug combinations, using a dedicated human 3D bone marrow system, comprising MSCs, osteoblasts and endothelial cells that allow maintenance of hematopoietic stem cells [28, 36]. Primary resistant AML mononuclear cells were introduced into the 3D system for one day, before adding AraC in combination with E6201 or verteporfin for 2 weeks. The leukemic cell microenvironment-bound fraction was then recovered and analyzed by flow cytometry (Fig. 6G). Analysis of the CD45+ leukemic fraction from the bone marrow niche showed that both E6201 and verteporfin significantly decreased the survival of AML drug-resistant cells (Fig. 6H, I), suggesting that targeting mechanotransduction pathways impairs chemoresistant leukemic cell growth in a bone marrow resident context.

## Discussion

AML patients have a high mortality rate, often relapse and display resistance to chemotherapy and to other targeted therapies. Here we show that AML-resistant cells display increased expression of mechanotransduction pathways that are associated with enhanced AML cell deformability and adhesion to their surrounding microenvironment.

Interestingly, it has been reported that selection of resistant clones upon treatment could be supported by improved fitness due to particular molecular abnormalities, but also by a better adhesion to the AML niche. Indeed, stroma-mediated chemoresistance in BM-resident leukemic cells is promoted by α4β1 integrins through activation of NF-κB [37, 38]. In addition, overexpression of METTL3 in idarubicin-resistant cells was reported to stabilize α4 integrin RNA and increase their capacity for migration and homing, both in vitro and in vivo [39]. Since leukemic cells, as well as hematopoietic cells, transit between endosteal and perivascular niches, we herein evaluated leukemic cell adhesion to substrates of various stiffnesses, correlated to corresponding niches [40]. Generally stiff substrates lead to a greater stretching of the cell than soft substrates [41] in epithelial and stromal cells, and were correlated with increased actin remodeling. However, for chemoresistant leukemic cells, we observed an enhanced adhesion and P-FAK activation on softer substrates, which could be due to the adaptation of more deformable cells to the rigidity of the substrate by mechano-reciprocity. It has been shown that the mean shear relaxation modulus increases upon cancer transformation in chronic myeloid leukemia (CML) [42], unlike epithelial cancers, suggesting that cell intrinsic stiffness is associated with different features in hematopoietic and epithelial solid cancers. Here, we demonstrated that AML chemo- or venetoclax- resistant cells are more deformable than AML-sensitive cells, suggesting that increased deformability could be a marker of treatment resistance. Dephosphorylation of Ezrin-Radixin-Moesin (ERM) proteins, linking transmembrane proteins to the actin cytoskeleton, is also associated with intrinsic cell stiffness and adhesion of hematopoietic cells [43], or leukemic cells [44]. AML chemoresistant cells displayed increased cytoskeleton network, as per F-actin staining, compared to AML-sensitive cells. While increased actin is usually associated with the ability to form focal adhesions, it is also frequently correlated to intrinsic cell stiffness [45]. Further studies would be required to investigate whether ERM proteins or other cytoskeletal molecules involved in cell stiffness, such as microtubules [46] or cholesterol [47], play a role in intrinsic cell deformability and adaptation to various soft/stiff microenvironments of treatment-resistant AML cells.

Our results suggest that BMP4 overexpression found in AML patient bone marrow plasmas [14] could partly be due to the confinement of MSCs and endothelial cells by the overexpansion of leukemic cells which accumulate in the limited space available in the BM. In CML, BMP4 regulates quiescence [12] and resistance by promoting the expression of TWIST-1 [13], while in AML, BMP4 was associated with the dedifferentiation of leukemic cells at diagnosis through BMPR1A [14]. In relapsed AML patients, BMPR1B overexpression in resistant leukemic cells promotes their activation by BMP4, in a different manner than in CML [13]. Indeed, we described here the induction of an ID1-specific expression in AML-resistant cells. Interestingly, a recent study showed that patients with AML subtype M2 or M5 with high ID1 expression had a significantly worse overall survival and shorter median survival than patients with low ID1 expression [48]. In addition, since inhibition of BMP signaling reduces β1-integrin activation in AML drug-resistant cells and is co-dependent on both integrin and stiffness [17, 49], further analyses should be conducted to determine if and how BMP4 loading on soft/stiff ECM could modulate AML cell adherence.

Another feature of AML-resistant lines and patients at relapse is the overexpression of TAZ /TEADs and CYR61. All three elements of the Hippo mechanical pathway are described for their oncogenic role in different cancers [21] and regulate cell polarization in the epithelium but also cell proliferation depending on the microenvironment. Pearson et al. highlighted so-called YAP^on^ or YAP^off^ cancers, where semi-solid cancers and leukemias fall into the second category. The re-expression of YAP in YAP^off^ cancers triggers the expression of integrins and ECM proteins, leading to cytostasis, mediated in part by αvβ5 integrins [23]. Studies in multiple myeloma also support this postulate in a hematopoietic context, with ectopic expression of YAP/TAZ in cells having an anti-tumor effect [24, 25]. Our results demonstrate that AML-sensitive cells do not express YAP at the protein level, and no or little TAZ and TEADs, while AML-resistant cells express more endogenous TAZ and TEADs, but not YAP. While YAP and TAZ display some common roles and share regulatory mechanisms, it has also been shown in epithelial cancers that they can behave differently, with YAP promoting increased expression of genes related to cell division and cell progression, whereas TAZ favors enhanced expression of genes associated with ECM organization and adhesion [50]. Interestingly, the particular induction of TAZ/TEAD described here echoes the specific activation of FLT3-TAZ signaling in blast phase CML [51], thus supporting that in the context of drug-resistant AML, TAZ/TEAD, but not YAP, are specifically overexpressed and improve cell adhesion in the leukemic niche.

Our study thus highlights the role of BMPR1B, and TAZ/TEADs expression in cells resistant to AraC and venetoclax, as well as their association with the adhesion of leukemic cells and their enhanced deformability. Moreover, the two pathways BMPR1B and TAZ/TEAD seem to be complementary. Acquiring BMPR1B expression would be an asset for resistant cells in order to sense their environment and to engage with BMP4 to induce the expression of ID1 and potentially promote quiescence or proliferation depending on its adhesion status. TAZ/TEAD4 may enhance cell survival, modulated by external and internal traction forces.

## Supplementary information

Supplementary informations

## Data Availability

Bulk RNAseq dataset is deposited at GEO under accession code GSE311903.
