# Supplementary material for: Targeting BMP and TAZ/TEAD mechanotransduction pathways impairs acute myeloid leukemia chemoresistance
Source: Leukemia. 2026 Mar 18;40(6):1151–62. doi: 10.1038/s41375-026-02904-7 (PMC13233328; doi:10.1038/s41375-026-02904-7)
Supplement: Supplementary file 1 — Supplementary informations [file 41375_2026_2904_MOESM1_ESM.pdf]

Targeting BMP and TAZ/TEAD mechanotransduction pathways impairs acute myeloid leukemia chemoresistance

Léa Barral[1,2,3], Nicolas Lespinasse[1,2,3], Camila Martin Cardozo[1,2,4], Sandrine Jeanpierre[1,2,3,5], Anna Bourgeois[1,2,3], Katharina Rösel[1,2,3], Emmanuel Beillard[5], Djohana Laurent[6], Pauline Peyrouze[6], Amine Belhabri[5], Yann Guillermin[5], Frederic Mazurier[7], Meyling Cheok[6], Marie-Charlotte Audry-Deschamps[4], Magalie Faivre[4], Véronique Maguer-Satta[1,2,3,4] and Sylvain Lefort[1,2,3,*].

1-CNRS UMR5286, Centre de Recherche en Cancérologie de Lyon, 69000 Lyon; 2-Inserm U1052, Centre de Recherche en Cancérologie de Lyon, 69000 Lyon; 3-Universite Claude Bernard Lyon 1, CRCL 69000, Lyon, France; 4- Univ Lyon, UCBL, INSA Lyon, ECL, CNRS, CPE Lyon, INL, UMR5270, 69622 Villeurbanne, France; 5-Centre Léon Bérard, 69000 Lyon; 6- CNRS-UMR9020, INSERM-U1366, University of Lille, Lille Hospital, CRC-Lille Cancer Research Center of Lille, Lille, France; 7- Univ. Rennes, CNRS, Inserm, IGDR (Institut de Génétique et Développement de Rennes), UMR6290, ERL U1305, 35000 Rennes, France.

* *Corresponding author:* Dr Sylvain LEFORT Cancer Research Center of Lyon-CRCL, U1052-UMR5286, 28 rue Laennec, 69373 Lyon Cedex 08, FRANCE, 33-478 782 907, Fax 33-478 782 907, sylvain.lefort@lyon.unicancer.fr

SUPPLEMENTARY METHODS

Pressure drop measurements

Device fabrication: Microfluidic channels in polydimethylsiloxane Sylgard 184 (PDMS) were manufactured using standard soft photolithographic techniques [1] and sealed on glass via oxygen plasma treatment (Harrick plasma, USA). The geometry consisted in two identical 100 μm wide channels funneling into a 7 μm wide and 200 μm long constriction. The two channels merged on the right side of the chip, and the distribution of the two fluids (co-flow virtual interface) was visualized using a contrast agent.

Cell samples: Cells were centrifuged at 1,000 *rpm* for 5 min and the supernatant was discarded before re-suspending the cells in RPMI 1640 with 10% FBS, 1% PS and 9.77 mg/mL dextran (Sigma, D5376-100G) at a concentration around 10^6 cells/μL. The dextran solution was used to avoid cell sedimentation in the reservoir and guaranteed the injection of a homogeneous concentration of cells over time. This solution also increased the hydrodynamic stress applied to the cells in the channel. Viscosity, pH and osmolarity of the solutions were 31.5 mPa.s, 7.4 and 300 mOsmol, respectively. A mixture of PBS 1X and black ink (Octopus Fluids GmbH) with 9.77 mg/mL dextran was used as contrast agent. Dextran solutions were always filtered at 0.2 μm immediately before the experiments.

Video-microscopy: Polyethylene (PE 20) tubes (Harvard Apparatus, USA) connected the solution reservoirs to the inlet hole in the device and the outlet hole of the waste reservoir. The solutions were injected into the microfluidic chip by a flow control system MFCS™-EZ (Fluigent, France) at a pressure of 100 mbar. The flow of individual cells in the sensing zone of the chip, as well as the associated deflection of the virtual interface were recorded using an inverted phase contrast microscope (DMI8 from Leica, Germany) with a 40x magnification, equipped with a high-speed camera (Miro Lab 310 from Phantom, USA). Experiments were performed at room temperature and the recordings were done within two hours after seeding the cells in the dextran medium.

Image Analysis: Movie/image post-processing was performed using a self-edited Matlab (RRID:SCR_001622) code to study cell dynamics and virtual interface deflection. Briefly, on

each image, the position of the virtual interface was determined as previously explained [2], allowing the correlation of the interface deflation with the position of the cell in the constricted area. Through appropriate calibration, the deflation of the interface can be converted into a pressure drop measurement.

Atomic force microscopy

Atomic force microscope (AFM MFP-3D Asylum Research, Oxford Instrument) was used to measure cell stiffness through force measurement between a soft pyramidal tip cantilever (PNP-TR, NanoWorld, knom = 0.08 N/m) and ML2 cells centrifugated at 1,000 rpm onto a poly-L-lysine covered glass slide in order to increase cell adherence. Cantilever stiffness was determined with the thermal noise method and resulted in a 0.025 N/m spring constant. Force measurements were made at a 200 nm/s approach velocity and a 2 µm/s retraction velocity on a 5 µm distance, 500 Hz sample rate and 2 nN setpoint. Measurements were carried out on 50 ML2-S and 50 ML2-R, in PBS medium at room temperature. The force-F vs indentation-δ part of the curve during approach was used to determine Young's modulus E following the suitable model for a pyramidal indenter:

$$F= (3E \tan(\theta))/4(1-v^2)\ \delta^2 \quad [4]$$

Where v is Poisson's ratio (fixed to 0.5) and θ is the pyramidal tip half-angle (35°).

RNA analysis

Transcript levels were analyzed either using normalized RNAseq expression values obtained from datasets of paired AML samples at diagnosis and relapse (GSE106291[3], OSHU[4]) or from horizontal meta-analysis that integrated transcriptomic data of AML from multicentric multiple studies (GSE147515, [5]).

RNA extraction was made using Trizol Reagent, and a DirectZol extraction kit (Zymo Research). cDNA library preparation and RNA sequencing were performed by Novogene using an Illumina NovaSeq 6000 sequencer in 150bp pair-end using Illumina PE150 technology and submitted to GEO (GSE311903). Normalization and differential expression analysis were performed with DESeq2 (V2.11.40.8) (parameters: fit type=parametric; Alpha

value for MA-plot=0.1). Volcano plots were generated with the package EnhancedVolcano. For each comparison, an unpaired two-tailed Student t test was performed. Genes were considered differentially expressed when their log2-transformed fold change was higher or lower than 0.5 and their p-value lower than 0.001.

Alternatively, reverse transcription using the SuperScript enzyme from the Takara kit was then performed, followed by qPCR using SyberGreen from a Takara kit as a fluorescent DNA intercalant, and amplicons were read on a Light Cycle 480 (Roche). Housekeeping genes such as HPRT (Hypoxanthine-Guanine-Phospho-Rybosyl-Transferase) and TBP (TATA-box Binding Protein) were used as reference for these analyses. Normalization was performed against healthy cytapheresis, and control cells, if necessary. The sequences of all primers are given in Supplementary Table S1.

SUPPLEMENTARY REFERENCES

1 Duffy DC, McDonald JC, Schueller OJ, Whitesides GM. Rapid Prototyping of Microfluidic Systems in Poly(dimethylsiloxane). *Anal Chem* 1998; **70**: 4974–4984.

2 Abkarian M, Faivre M, Stone HA. High-speed microfluidic differential manometer for cellular-scale hydrodynamics. *Proc Natl Acad Sci U S A* 2006; **103**: 538–542.

3 Shlush LI, Mitchell A, Heisler L, Abelson S, Ng SWK, Trotman-Grant A *et al.* Tracing the origins of relapse in acute myeloid leukaemia to stem cells. *Nature* 2017; **547**: 104–108.

4 Tyner JW, Tognon CE, Bottomly D, Wilmot B, Kurtz SE, Savage SL *et al.* Functional Genomic Landscape of Acute Myeloid Leukemia. *Nature* 2018; **562**: 526–531.

5 Nehme A, Dakik H, Picou F, Cheok M, Preudhomme C, Dombret H *et al.* Horizontal meta-analysis identifies common deregulated genes across AML subgroups providing a robust prognostic signature. *Blood Adv* 2020; **4**: 5322–5335.

HPRT FW	TGACCTTGATTTATTTTGCATACC
HPRT REV	CGAGCAAGACGTTCAGTCCT
TBP FW	CACGAACCACGGCACTGATT
TBP REV	TTTTCTTGCTGCCAGTCTGGAC
ID1 FW	GTTACTCACGCCTCAAGGAGC
ID1 REV	AGAAGAAATGAGACCGGCGGG
TEAD4 FW	GCCTTCCACAGTAGCATGG
TEAD4 REV	AAAGCTCCTTGCCAAAACC
YAP1 FW	ACAATGACGACCAATAGCTCAGAT
YAP1 REV	AACGGTTCTGCTGTGAGGG3
TAZ FW	CCCTCATCACCGTGTCCAA
TAZ REV	ACGCATCAACTTCAGGTTCCA
BMP2 FW	ACGCTCTTTCAATGGACGTG
BMP2 REV	GGAAGCAGCAACGCTAGAAG
BMP4 FW	CTTTACCGGCTTCAGTCTGG
BMP4 REV	GGGATGCTGCTGAGGTTAAA
BMPR1B FW	CTGTGGTCACTTCTGGTTGC
BMPR1B REV	TTCCTTTCTGTGCAGCATTC
CTGF FW	GGAGTGGGTGTGTGACGAG
CTGF REV	CTTCCAGTCGGTAAGCCGC
Cyr61 FW	CAGGACTGTGAAGATGCGGT
Cyr61 REV	GCCTGTAGAAGGGAAACGCT

Table S1. List of primers used for RT-QPCR

Supp Figure 1

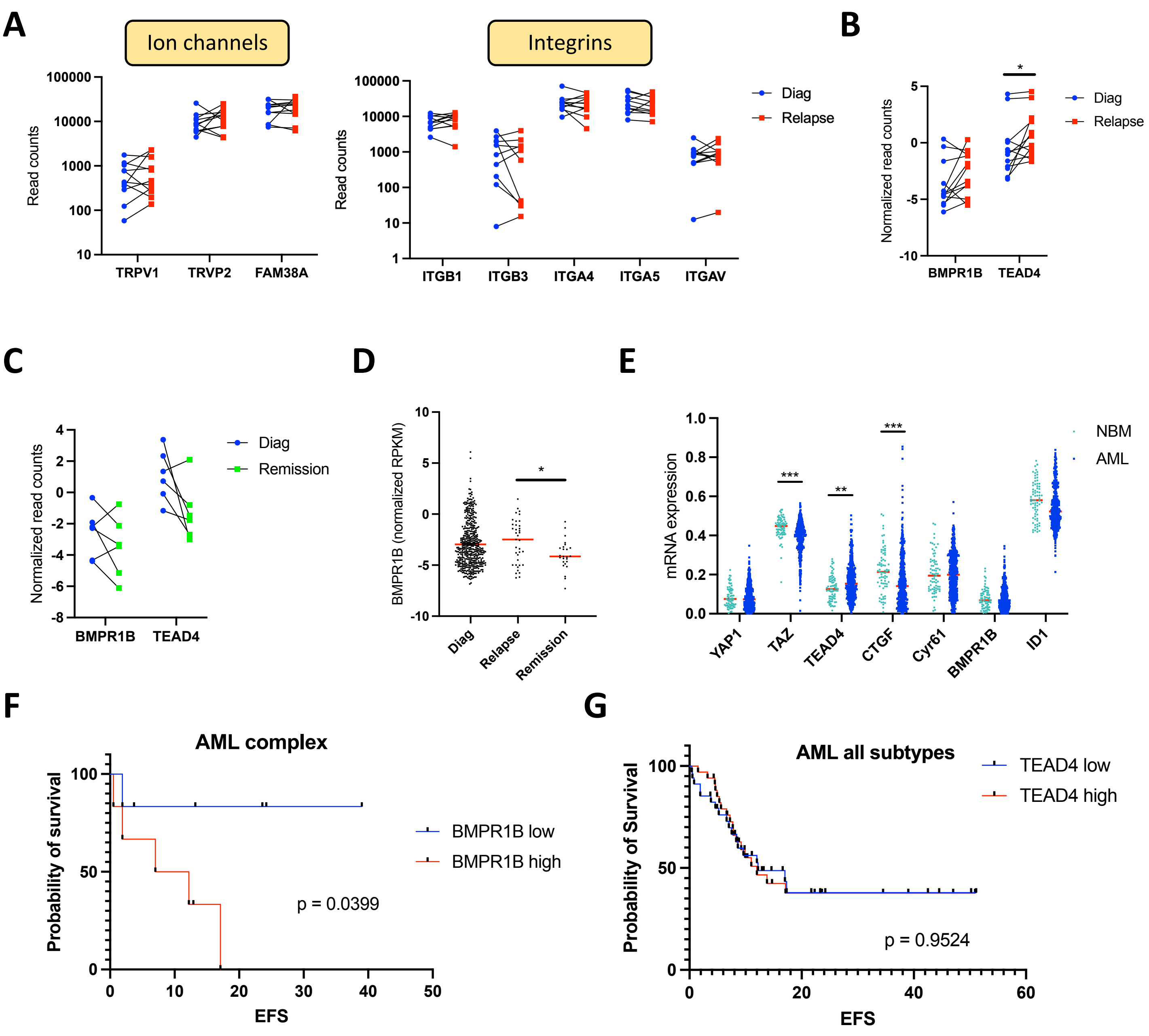

Supplementary Fig. 1. AML relapsed patients display increased BMP and Hippo elements. A, Relative mRNA expression from paired AML primary samples at diagnosis and relapse (GSE106291), represented as Ions channels family (left panel; TRVP1, TRVP2, FAM38A), integrin family (right panel; ITGB1, ITGB3, ITGA4, ITGA5, ITGAV). Values are shown as RPKMs and represent 11 patients for each gene. **B-C,** Relative BMPR1B and TEAD4 mRNA expression from paired AML primary samples at diagnosis and relapse (**B**) or remission (**C**) (OSHU). Values are shown as RPKMs and represent 12 patients (relapse) or 6 patients (remission) for each gene. **D,** mRNA expression of BMPR1B from AML samples at diagnosis (n=450), relapse (n=37) or remission (n=25) stages (OSHU). Values are shown as normalized RPKMs. **E,** mRNA expression of YAP1, TAZ, TEAD4, CTGF, CYR61, BMPR1B and ID1 in normal bone marrow (NBM) or AML bone marrow (GSE147515). **F-G** Kaplan-Meier curves of event-free survival from GSE147515 in complex AML karyotypes (n=12) with respect to BMPR1B (**F**) or in all AML subtypes with respect to TEAD4 (**G**) mRNA level (N = 68).

Supp Figure 2

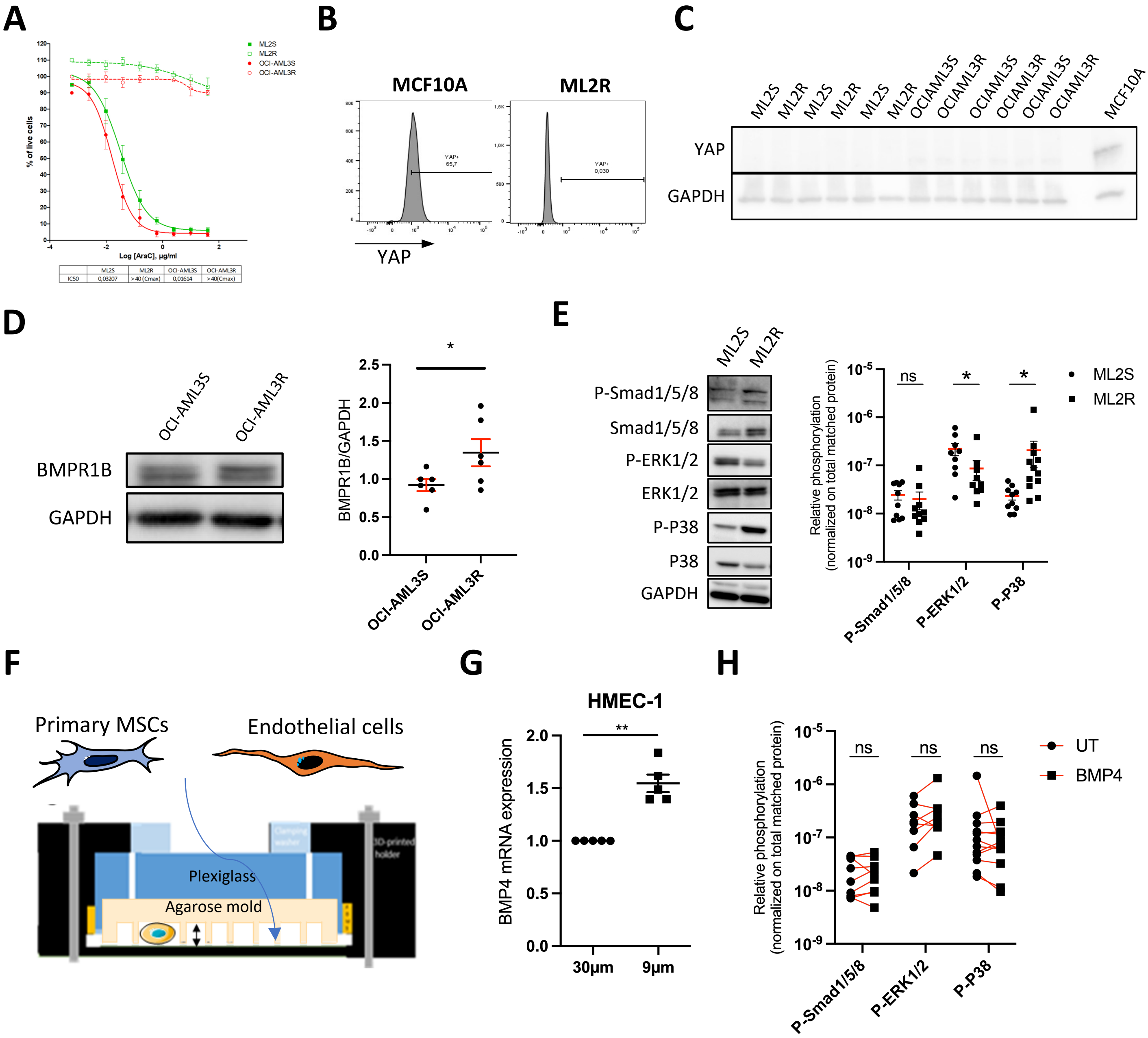

Supplementary Fig. 2. TEAD and BMP4-BMPR1B axis are overexpressed and active in AML chemoresistant models. A, IC50 of chemo-sensitive or – resistant ML2 or OCI-AML3 upon Ara-C treatment. **B,** Representative picture of YAP staining from MCF10A (left panel) or ML2R (right panel) cells. **C,** Western blots showing YAP levels (relative to GAPDH) of Ara-C-sensitive (S) or Ara-C-resistant (R) ML2 or OCI-AML3 or MCF10A cells. **D,** Western blots showing BMPR1B levels (relative to GAPDH) of Ara-C-sensitive (S) or Ara-C-resistant (R) OCI-AML3 cells grown for 24 h. Individual data are from independent experiments. **E,** Western blots showing P-Smad1/5/8, P-Smad1/5/8, P-ERK1/2, ERK1/2, P-P38, P38 levels (relative to GAPDH) of Ara-C sensitive (S) or Ara-C resistant (R) ML2 cells grown for 24 h. Individual data are from independent experiments. **F,** Representative picture of the confinement system, where primary MSC or endothelial were cultured for 72 h. **G,** BMP4 mRNA expression in HMEC cells after 72h of confinement at 9µm, normalized to cells confined at 30µm. **H,** Dot plot showing phosphorylation levels (normalized on total levels) in Ara-C sensitive ML2 cells +/- BMP4. Individual data are from independent experiments.

Supp Figure 3

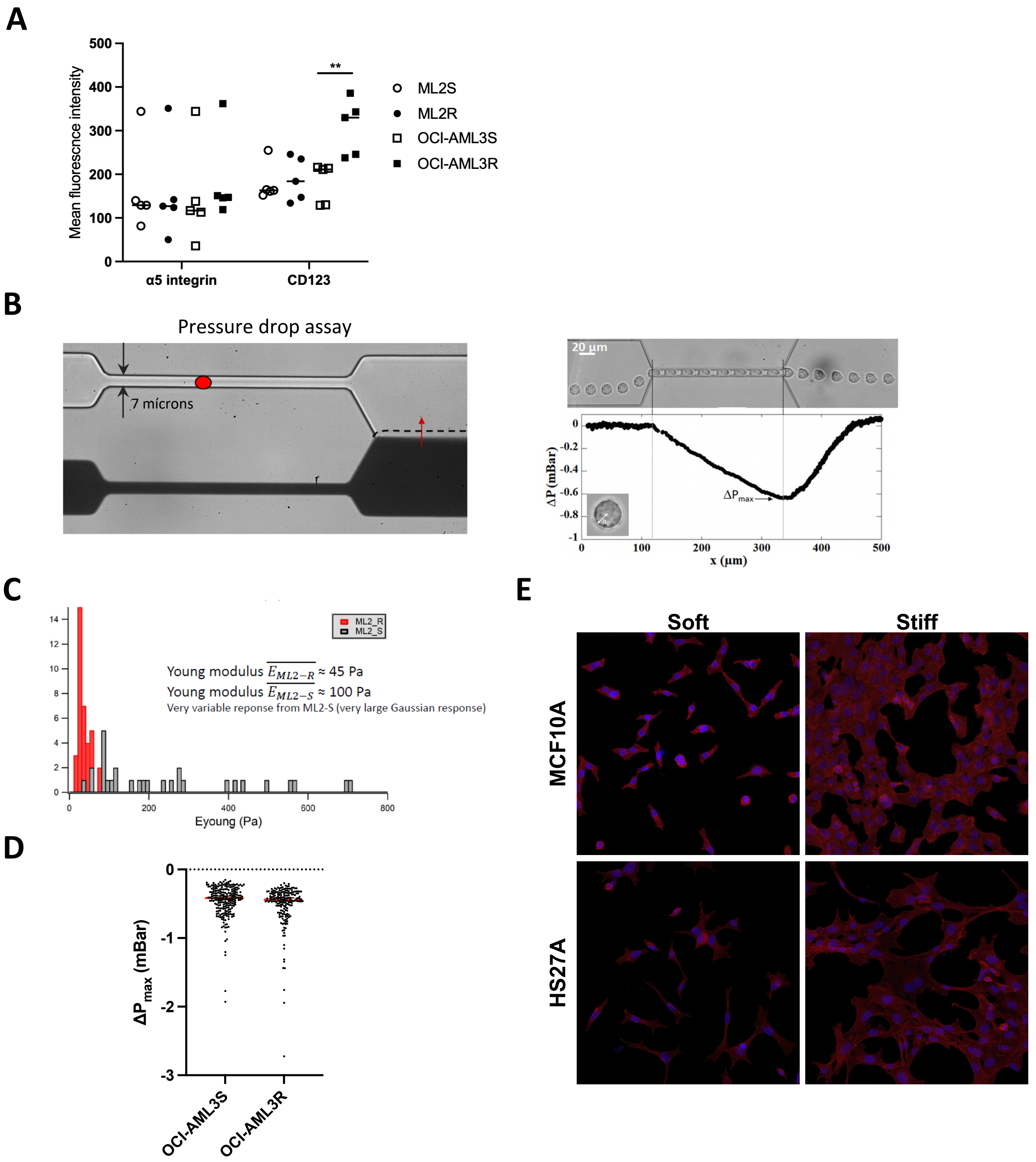

Supplementary Fig. 3. Increased cell deformability promotes adhesion of chemoresistant AML cells on softer substrates. A, Dot plot showing MFI of α5-integrin and CD123 in Ara-C sensitive (open circles/squares) or Ara-C resistant (filled circles/squares) cells. Individual data are from independent experiments. **B,** Representative picture of Pressure-drop microfluidic system, showing the deflection of the interface when cells go through upper constriction. **C,** Graph representing ML2S (grey) or ML2R (red) Young's modulus measured by atomic force microscopy. **D,** Dot plot representing the maximum pressure drop of OCI-AML3S and OCI-AML3R. Individual dot represent a single value per cell (from 4 independent experiments). **E,** Representative pictures of F-actin and P-FAK staining from MCF10A (Top panel) or HS27A (bottom panel) cells coated on soft (0.5kPa) or stiff (4kPa) hydrogels.

Supp Figure 4

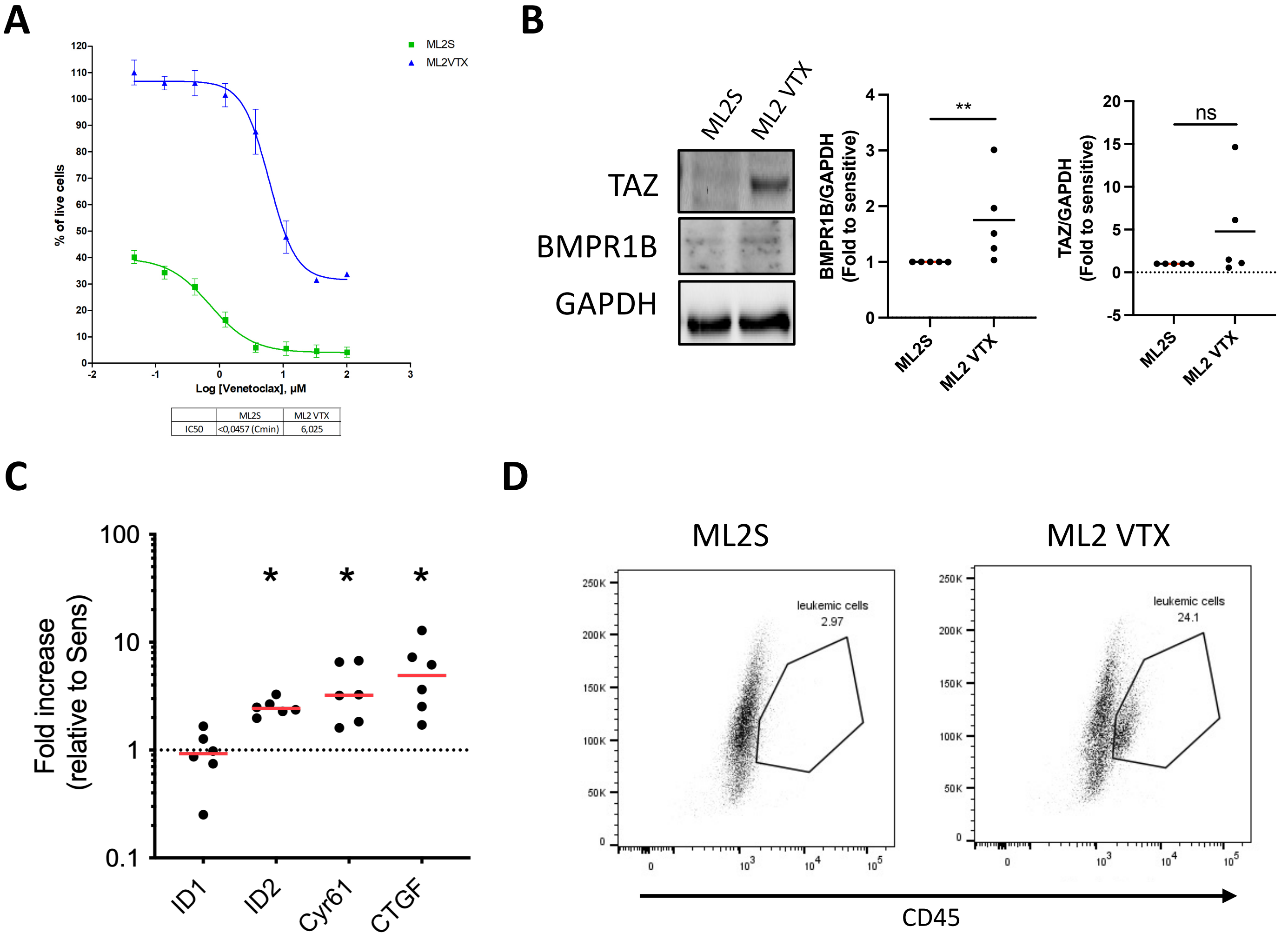

Supplementary Fig. 4. Venetoclax resistant AML cells also display high levels of BMPR1B and TEAD levels, associated to enhanced adhesion and increased deformability. A, IC50 of Venetoclax-sensitive or – resistant ML2 upon Venetoclax treatment. **B,** Western blots showing TAZ and BMPR1B levels (relative to GAPDH) of venetoclax-sensitive (S) or -resistant (VTX) ML2 cells grown for 24 h. Individual data are from independent experiments. **C,** mRNA expression of ID1, ID2, CYR61 and CTGF from Venetoclax resistant ML2 cells normalized to Venetoclax sensitive cells ± SEM. Individual data are from independent experiments. **D,** Representative pictures of CD45 staining from 24h co-culture of HS27A with ML2S (left panel) or ML2 VTX (right panel).

Supp Figure 5

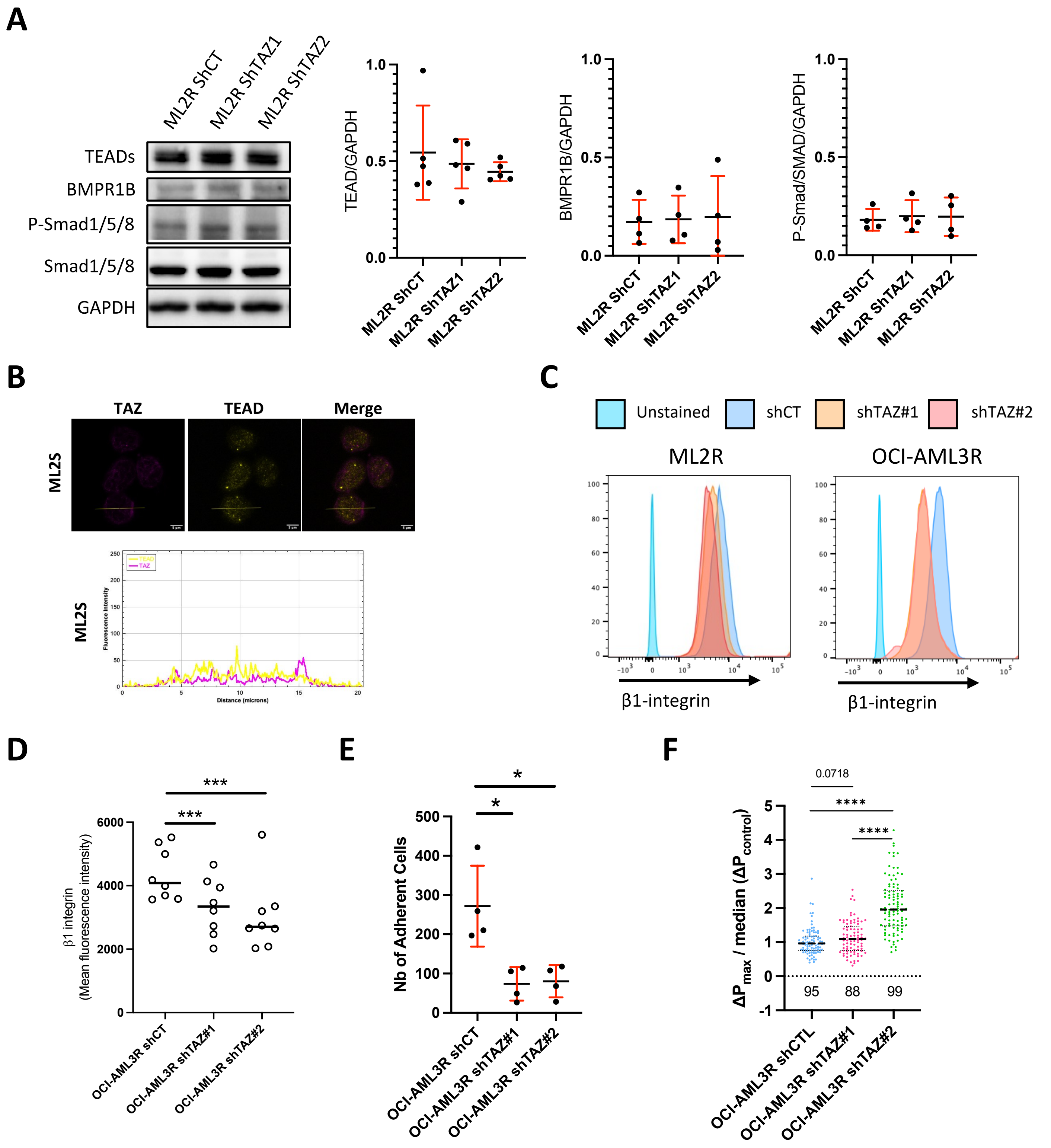

Supplementary Fig. 5. Targeting the Hippo pathway leads to alterations in the biomechanical properties of chemoresistant AML cells. A, Western blots showing TEAD, BMPR1B, P-SMAD1/5/8, SMAD1/5/8 levels (relative to GAPDH) of Ara-C-resistant (R) ML2 cells harboring shCtl, shTAZ#1 or shTAZ#2. **B,** Representative images of TAZ and TEAD staining from ML2S cells. Scale bar, 5 µm. Graph showing Line profile colocalization analysis from ML2S cells **C,** Staining intensity of β-integrin expression in ML2R and OCI-AML3R cells. **D,** Dot plot showing Mean Fluorescent Intensity (MFI) of β-integrin in Ara-C-resistant OCI-AML3R cells harboring shCtl, shTAZ#1 or shTAZ#2. Individual data are from independent experiments. **E,** Dot plot showing the number of adherent cells for OCI-AML3R cells harboring shCtl, shTAZ#1 or shTAZ#2. Individual data are from independent experiments. **F,** Dot plot representing the maximum pressure drop of OCI-AML3R cells harboring shCtl, shTAZ#1 or shTAZ#2. Individual dot represent a single value per cell (from 3 independent experiments).

Supp Figure 6

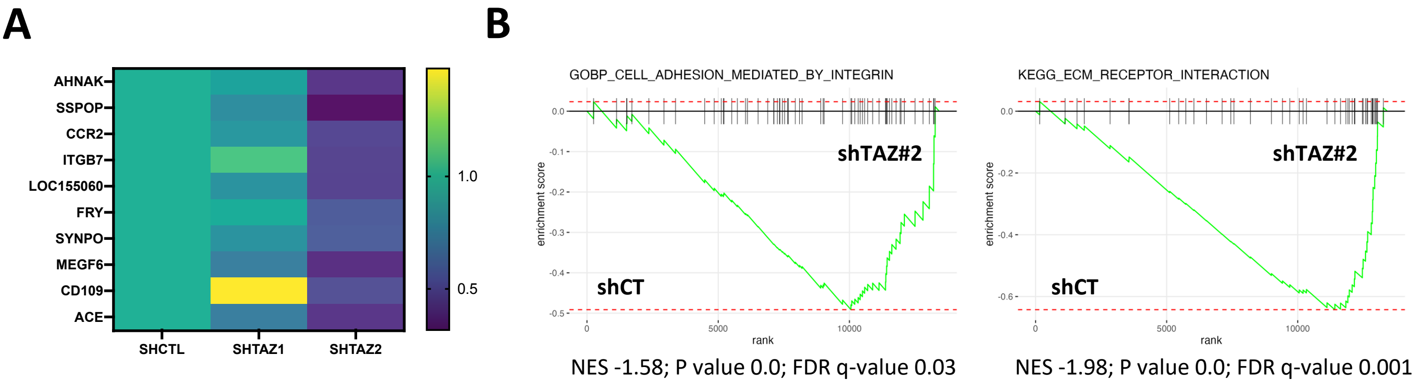

Supplementary Fig. 6. Targeting the Hippo pathway of chemoresistant AML cells leads to alterations of cell adhesion signatures. A, Expression of most differentially expressed genes from ML2R cells harboring shCtl, shTAZ#1 or shTAZ#2. **B,** GSEA of shCtl versus shTAZ#2 ML2R cells. Gene sets shown are cell adhesion mediated by integrin (left) and ECM receptor interaction-associated genes.

Supp Figure 7

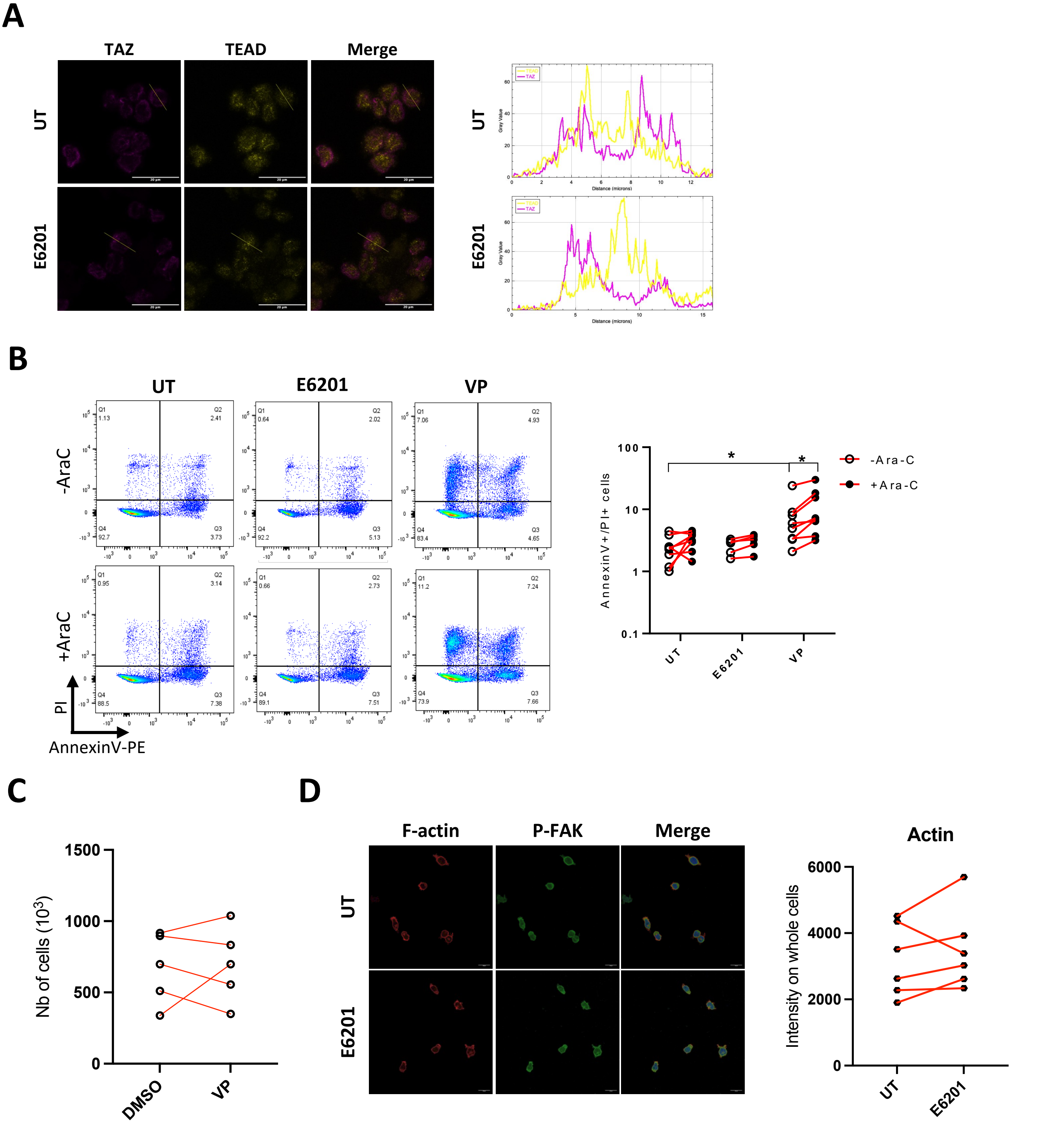

Supplementary Fig. 7. Targeting BMPR1B or TAZ/TEAD impair AML bone marrow persistence. A, Representative images of TAZ and TEAD staining from Ara-C-resistant ML2 cells untreated or treated with E6201 for 24 h. Scale bar, 20 µm. Graph showing Line profile colocalization analysis from UT or E6201-treated ML2R cells. **B,** Representative plots of apoptosis staining by annexinV/PI from Ara-C-resistant ML2 (R) after 48 h with no treatment (UT), with E6201 (100 nM) or VP (0.25 µM), in combination with AraC (1 µM). Dot plot showing MFI of annexinV+/PI+ cells from Ara-C-resistant ML2 cells treated with E6201, VP +/- Ara-C. **C,** Number of ML2R cells after 72 h culture, treated with DMSO or VP (0.25 µM). **D,** Representative images of F-actin and P-FAK staining from Ara-C-resistant ML2 cells treated with E6201 for 24 h. Scale bar, 30 µm. Dot plot showing F-actin intensity from untreated or E6201-treated ML2R cells.

Supp Figure 8

A

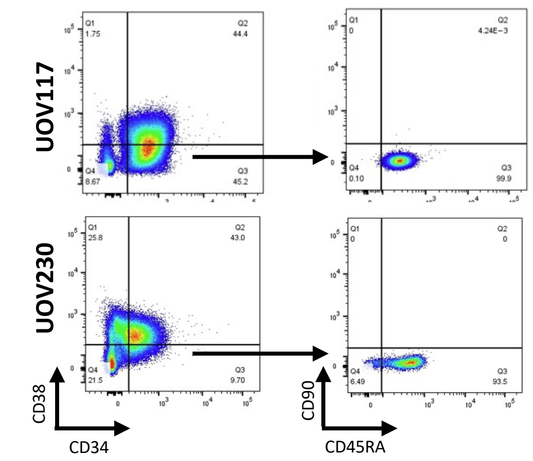

B

	Sex	Age	Score ELN2022	Fusion	Mutations
**UOV34**	F	39	Favorable	*CBFB (ex.5)::MYH11 (ex.3)*	NRAS
**UOV72**	F	34	Adverse	*KMT2A (ex.8)::AFDN (ex.2)*	JAK3, SF3B1
**UOV117**	M	49	Adverse		JAK3, U2AF1, ETV6
**UOV148**	M	30	Intermediate		FLT3, DNMT3A, NPM1
**UOV162**	F	49	Intermediate	*FLT3-ITD (21 pb)*	NRAS, SF3B1
**UOV230**	F	49	Adverse		RUNX1
**UOV324**	F	60	Favorable	*CEBPA-ITD (87 pb)*	IDH2, DNMT3A
**UOV499**	M	63	Adverse		NRAS,U2AF1, DNMT3A

C

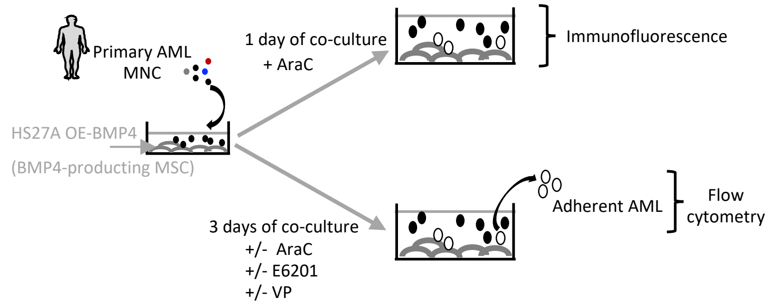

D

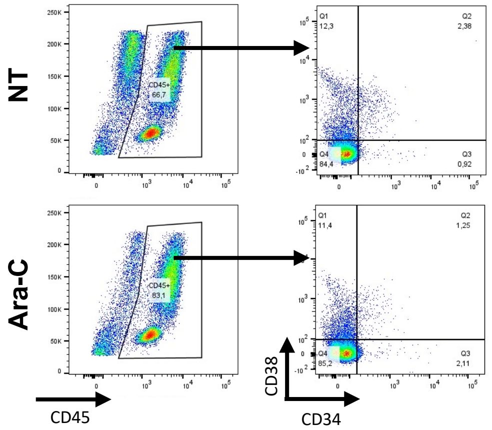

E

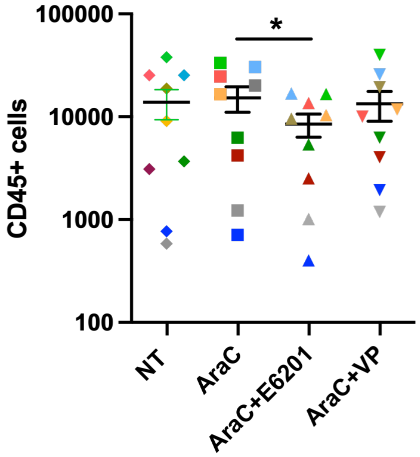

Supplementary Fig. 8. Targeting BMPR1B or TAZ/TEAD impairs AML bone marrow persistence. A, Representative pictures of CD34/CD38 and CD45RA/CD90 staining from primary resistant AML cells. **B,** Table showing clinical and molecular features of primary resistant AML cells. **C,** Experimental schematic diagram of primary AML relapse samples co-culture on BMP4 overexpressing HS27A, with the combination of Ara-C and E6201 or VP for 1 or 3 days. **D,** Representative pictures of CD45 and CD34/CD38 staining from primary resistant AML cells after 3 days of co-culture with HS27A OE-BMP4. **E,** Dot plot showing the number of CD45+ cells from 3 days co-culture. Each colored dot represents different primary AML relapsed patients
